# Estimating the age of Hb G‐Coushatta [β22(B4)Glu→Ala] mutation by haplotypes of β‐globin gene cluster in Denizli, Turkey

**DOI:** 10.1002/mgg3.404

**Published:** 2018-05-01

**Authors:** Onur Ozturk, Sanem Arikan, Ayfer Atalay, Erol O. Atalay

**Affiliations:** ^1^ Department of Biophysics Inonu University School of Medicine Malatya Turkey; ^2^ Department of Biophysics Pamukkale University School of Medicine Denizli Turkey

**Keywords:** β‐globin gene, haplotype, Hb G‐Coushatta, mutation age estimate, population genetic structure

## Abstract

**Background:**

Hb G‐Coushatta variant was reported from various populations’ parts of the world such as Thai, Korea, Algeria, Thailand, China, Japan and Turkey. In our study, we aimed to discuss the possible historical relationships of the Hb G‐Coushatta mutation with the possible migration routes of the world. For this purpose, associated haplotypes were determined using polymorphic loci in the beta globin gene cluster of hemoglobin G‐Coushatta and normal populations in Denizli, Turkey.

**Methods:**

We performed statistical analysis such as haplotype analysis, Hardy–Weinberg equilibrium, measurement of genetic diversity and population differentiation parameters, analysis of molecular variance using *F*‐statistics, historical‐demographic analyses, mismatch distribution analysis of both populations and applied the test statistics in Arlequin ver. 3.5 software program.

**Results:**

The diversity of haplotypes has been shown to indicate different genetic origins for two populations. However, AMOVA results, molecular diversity parameters and population demographic expansion times showed that the Hb G‐Coushatta mutation develops on the normal population gene pool. Our estimated τ values showed the average time since the demographic expansion for normal and Hb G‐Coushatta populations ranged from approximately 42,000 to 38,000 ybp, respectively.

**Conclusion:**

Our data suggest that Hb G‐Coushatta population originate in normal population in Denizli, Turkey. These results support the hypothesis that the multiple origin of Hb G‐Coushatta and indicate that mutation may have been triggered the formation of new variants on beta globin haplotypes.

## 1 INTRODUCTION

Hb G‐Coushatta [β22 (B4) Glu→Ala] (HGVS Name: *HBB*: c.68A>C) was first identified in American Coushatta Indians (Schneider, Haggard, McNutt, & Johnson, 1964). This abnormal hemoglobin variant was reported from various populations parts of the world such as Thai, Korea, Algeria, Thailand, China, Japan and Turkey (Atalay et al., 2005; Chinchang & Viprakasit, 2007; Dinçol, Dinçol, & Erdem, 1989; Itchayanan, Svasti, Srisomsap, Winichagoon, & Fucharoen, 1999; Sözmen, Uysal, & Akar, 1990; Yenice et al., 2000). Beta globin gene cluster haplotypes are frequently encountered in population surveys. Human β‐globin gene cluster is located at chromosome 11 (5′‐ε‐G_γ_‐A_γ_‐ψβ‐δ‐β‐3′). The haplotypes obtained by testing seven polymorphic restriction sites in this region of the beta globin gene cluster provide important data on the structure of populations, their origins and their possible associations with mutations (Alcantara et al., 2003; Chen, Easteal, Board, & Kirk, 1990; Currat et al., 2002; De Lugo, Rodriguez‐Larralde, & De Guerra, 2003; Mattevi et al., 2000). Although data on the β‐globin gene cluster haplotypes are limited for the world cases, there are four different suggested genetic origins of these haplotypes reported in association with the Hb G‐Coushatta cases American Indian [− + − − + − ?], Chinese [− + + − + + ?], Kocaeli‐Turkey [− + + − − − +], and Denizli‐Turkey [− + − + + + +] (Li et al., 1999; Ozturk et al., 2007). In our previous studies based on halotype analysis for the abnormal hemoglobins detected in Denizli‐Turkey, the average time since the demographic expansion of Hb D‐Los Angeles population was calculated as ranged from approximately 38,000 (95% CI 18,500–62,000) ybp, Hb S population 26,000 ybp (95% CI; 11,000–36,000) and the normal population 42,000 (95% CI, 25,000–58,000) ybp, respectively (Ozturk, Arikan, Atalay, & Atalay, 2016, 2017).

In our study, we aimed to discuss the possible historical relationships of the Hb G‐Coushatta mutation with the possible migration routes of the world. In accordance for this purpose we tested the Hb G‐Coushatta population and the normal population haplotype data in the Denizli region comparatively with the statistical software program Arlequin ver 3.5 (Excoffier, Laval, & Schneider, 2005; Excoffier & Lischer, 2010). Associated haplotypes were determined using polymorphic loci in the β‐globin gene cluster of both populations.

## 2 MATERIALS AND METHODS

### 2.1 Sample collection

We studied 15 unrelated patients with abnormal Hb G‐Coushatta and 59 unrelated normal DNA samples. It has been reported in previously published articles that during the identification of these haplotype data were used 59 unrelated healthy subjects DNA samples (Ozturk et al., 2016). Normal and Hb G‐Coushatta DNA samples were taken from Pamukkale University, Medical Faculty, Department of Biophysics DNA Bank (Denizli, Turkey) as anonymous samples. Written informed consent has been already taken from individuals and/or from their parents for further anonymous DNA analysis.

### 2.2 Haplotype identification and statistical analysis

In the first step of our study, PCR‐RFLP (Polymerase chain reaction‐restriction fragment length polymorphism) method was applied on seven polymorphic restriction sites (HincII 5′ to ε, HindIII 5′ to Gγ, HindIII in the IVS‐II 5′ to Aγ, HincII in ψβ, HincII 3′ to ψβ, AvaII in β, HinfI 3′ to β) in the β‐globin gene cluster as previously reported (Ozturk et al., 2016). Associated haplotypes for the normal population samples and patients with Hb G‐Coushatta were determined by the obtained RFLP results. We performed statistical analysis of both populations and applied the test statistics in Arlequin 3.5 software program with unknown gametic phase such as haplotype analysis (Excoffier et al., 2005; Falchi et al., 2005), Hardy–Weinberg equilibrium tests (Excoffier & Lischer, 2010; Excoffier et al., 2005) measurement of genetic diversity and population differentiation parameters, analysis of molecular variance (AMOVA) using *F*‐statistics (*F*_ST_, *F*_IT_, *F*_IS_) (Mantel, 1967; Schneider, Roessli, & Excoffier, 2000; Slatkin, 1995; Wright, 1965), historical‐demographic analyses (Tajima's Fu's tests) (Fu, 1997; Tajima, 1989a), mismatch distribution analysis, analyses of tau (τ) and initial theta, SSD, the Harpending's raggedness index (Hri) and *p*‐values of SSD (Excoffier, 2004; Harpending, 1994; Ray, Curratand, & Excoffier, 2003; Rogers, 1995; Rogers & Harpending, 1992; Schneider & Excoffier, 1999; Slatkin & Hudson, 1991) as previously reported (Ozturk et al., 2016). The Rogers and Harpending (1992) model was used to calculate the time elapsed since the population expansion by estimating Tau (τ), θ_0_, and θ_1_ based on the mismatch distribution outputs from Arlequin. Historic demographic expansions were also investigated by the examination of frequency distributions of pairwise differences between sequences (mismatch distribution), which is based on three parameters: θ_0_, θ_1_ (θ before and after the population growth) and τ (time since expansion expressed in unit of mutational time).

## 3 RESULTS

Tables 1 and 2 show the summary of listed frequencies and haplotypes of Hb G‐Coushatta and normal populations respectively. In normal population the haplotype with the highest frequency is Mediterranean haplotype I [+ − − − − + +] (14%). However, in Hb G‐Coushatta population the Mediterranean haplotype I [+− – – – + +] does not have any frequency value.

**Table 1:** β‐globin gene cluster haplotypes for the seven loci in association with the Hb G‐Coushatta [β22(B4)Glu→Ala] (HGVS Name: *HBB*:c.68A>C) population in Denizli, Turkey

No.	Haplotype	Frequency	*SD*
1	+ + − + + + +	0.266667	0.082118
2	− − − − − + +	0.200000	0.074278
3	− + + + + + +	0.133333	0.063124
4	− + − + + + +	0.066667	0.046321
5	− − − − − + −	0.066667	0.046321
6	− + − − + + +	0.066667	0.046321
7	− + − − − + −	0.033333	0.033333
8	− − − − − − +	0.033333	0.033333
9	− + − − − + +	0.033333	0.033333
10	− + − + + − +	0.033333	0.033333
11	− + − − + + −	0.033333	0.033333
12	+ + − + + + −	0.033333	0.033333

Maximum‐likelihood haplotype frequencies generated by Arlequin 3.5 software.

Sum of 12 listed frequencies: 1.000000/No. of gene copies in sample: 30/*SD*: standard deviation.

**Table 2:** β‐globin gene cluster haplotypes for the seven loci in association with Normal population in Denizli, Turkey

No.	Haplotype	Frequency	*SD*
1	+ − − − − + +	0.144068	0.032465
2	+ + − + + + +	0.127119	0.030796
3	− + − + + + +	0.084746	0.025748
4	+ − − − − + −	0.076271	0.024539
5	− − − − − − −	0.067797	0.023242
6	− − − − − − +	0.059322	0.021839
7	− − − − − + +	0.050847	0.020310
8	+ + + − + + +	0.050847	0.020310
9	− + + − + + +	0.033898	0.016730
10	+ − − − − − −	0.033898	0.016730
11	− − − − − + −	0.033898	0.016730
12	+ + + + + + +	0.025424	0.014552
13	− + + + + + +	0.016949	0.011934
14	+ + − − + + +	0.016949	0.011934
15	− + − + + − −	0.016949	0.011934
16	− + − − − − −	0.016949	0.011934
17	+ − − − + + +	0.016949	0.011934
18	+ + − + + + −	0.016949	0.011934
19	+ − − − − − +	0.016949	0.011934
20	− + + − − − +	0.008475	0.008475
21	− + − − − − +	0.008475	0.008475
22	+ + − − − − +	0.008475	0.008475
23	+ − − − + + −	0.008475	0.008475
24	− + + + + + −	0.008475	0.008475
25	− + − − + − +	0.008475	0.008475
26	+ + − + + − +	0.008475	0.008475
27	− + − − + − −	0.008475	0.008475
28	− + − + + − +	0.008475	0.008475
29	− + − + + + −	0.008475	0.008475
30	+ + + − + − +	0.008475	0.008475

Maximum‐likelihood haplotype frequencies generated by Arlequin 3.5 software.

Sum of 30 listed frequencies: 1.000000/No. of gene copies in sample: 118/*SD*: standard deviation.

We tested the genetic differentiation of normal and Hb G‐Coushatta populations using the analysis of molecular variance (AMOVA) with Arlequin ver 3.5 (Excoffier, Smouse, & Quattro, 1992) (Table 3).

**Table 3:** (AMOVA) *F*‐statistics calculated for seven loci differentiation among populations of between Normal and Hb G‐Coushatta

		Distance method: Pairwise difference		
Source of variation	*df*	Sum of squares	Variance components	Percentage of variation
Among populations	1	5.817	0.09287 Va	5.96
Among individuals within populations	72	98.940	−0.08994 Vb	−5.78
Within individuals	74	115.000	1.55405 Vc	99.81
Total	147	219.757	1.55698	
Fixation Indices: Significance tests (1023 permutations)				
*F*_IS_: −0.06143	*p*‐value = .86413 ± .00941			
*F*_ST:_ 0.05964	*p*‐value = .01369 ± .00367			
*F*_IT_: 0.00188	*p*‐value = .73314 ± .01252			

Non‐differentiation: Exact *p* value is calculated by based on haplotype frequencies and controlled by Markov method.

Fixation indices (*F*_IS_, *F*_ST_ and *F*_IT_) *p* values calculated by Global test of differentiation among populations method. (Insignificant *p *> .05, significant *p *≤ .05).

Va means, We test *F*_ST_ by permuting haplotypes among populations.

Vb means, We test *F*_IS_ by permuting haplotypes among individuals within populations.

Vc means, We test *F*_IT_ by permuting haplotypes among individuals among populations.

**Figure 1:**
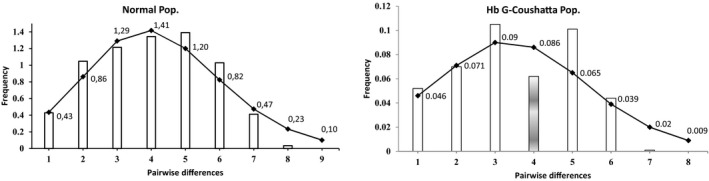
The observed pairwise difference (bars) and the expected mismatch distributions (solid line) under the sudden expansion model of Normal and Hb G‐Coushatta populations

We showed the summary of molecular diversity parameters for each population (Table 4), statistical demographic parameters for two populations with the mismatch distribution graphics (Figure 1), parameters of the graphic shape testing results Harpending's raggedness index and *p*‐values of sum of square deviations (SSD) (Table 5), respectively.

**Table 4:** Summary of molecular diversity for two populations

Populations	*n*	No. of haplo.	*k*	θ_S_	*h*	π	Tajima's *D*		Fu's *F*_S_		Mismatch distribution		
							*D*	*p*	*F*_S_	*p*	τ (95% CI)	θ_0_	θ_1_
Normal	59	30	3.03 ± 1.59	1.30 ± 0.56	0.93 ± 0.00	0.43 ± 0.25	3.00	.99	−16.88	.00	3.46 (4.48–0.91)	0.01	25.82
Hb‐G Coushatta	15	12	2.51 ± 1.39	1.76 ± 0.83	0.88 ± 0.03	0.35 ± 0.22	1.25	.86	−4.13	.02	3.14 (5.16–0.73)	0.00	12.01

Number of individuals (*n*), number of haplotype, average pairwise differences among individuals (*k*), number of segregating sites (*S*), haplotype diversity (*h *± standard deviation), nucleotide diversity (π ± standard deviation) for each populations. Tajima's *D* and Fu's *F*_S_, corresponding *p*‐value, and mismatch distribution parameter estimates for each population. *D* Tajima's *D* estimate population expansion, *F*_s_ Fu's *F*_s_ estimate population expansion. Values for τ, θ_0_, and θ_1_ are the age of the expansion, the population size before the expansion, and the population size after expansion, respectively, all expressed in units of mutation time. *Insignificant p *>* *0.05, significant *p *≤* *0.05. Tajima's *D* and Fu's *F*_s_, corresponding *p* values, mismatch distribution parameter estimates and error estimates for populations are ±standard deviation as calculated by Arlequin.

**Table 5:** Values of the mismatch distribution test statistics SSD and rg against a null hypothesis of population expansion

Goodness‐of‐fit tests				
Populations	SSD	SSD‐*p* value	rg	rg‐*p* value
Normal	0.00352	.190	0.02279	.590
Hb‐G Coushatta	0.01343	.160	0.05294	.290

SSD, sum of squared deviations; rg, Harpending's raggedness.

*p* (SSD) is the probability of observing by chance a less than good fit between the observed and mismatch distribution for a demographic history of the population defined by the estimated parameters τ, θ_0_, and θ_1._

In terms of the time estimations, parameter values of τ and historical population parameters θ (θ_0_ and θ_1_) also show a similar historical growth period for the populations (Table 4). The mean population age for normal and Hb G‐Coushatta populations in Denizli depends on results of estimations parameter values of τ, dated approximately 42,000 ybp (95% CI; 11,000–55,000) to 38,000 ybp (95% CI; 10,000–62,000), respectively (Table 4). The results in Table 6 show that normal population was in Hardy–Weinberg equilibrium (*p *> .05) for each of the seven polymorphic loci and the Hb G‐Coushatta population was in Hardy–Weinberg equilibrium except for fourth loci.

**Table 6:** Hardy–Weinberg equilibrium (HWE) test for all Loci in Normal and Hb‐G Coushatta populations

	Locus	#Genot	Obs.Het.	Exp.Het.	*p*‐value	*SD*	Steps done
Normal Pop.	1	59	0.44068	0.49718	.43526	0.00049	1001000
	2	59	0.44068	0.50413	.43380	0.00050	1001000
	3	59	0.23729	0.26076	.60583	0.00048	1001000
	4	59	0.37288	0.44039	.24685	0.00041	1001000
	5	59	0.57627	0.50297	.30238	0.00042	1001000
	6	59	0.49153	0.40635	.18981	0.00039	1001000
	7	59	0.45763	0.42083	.54962	0.00049	1001000
Hb G‐Coushatta Pop.	1	15	0.60000	0.43448	.23723	0.00043	1001000
	2	15	0.60000	0.43448	.23763	0.00041	1001000
	3	15	0.26667	0.23908	1.00000	0.00000	1001000
	4	15	0.93333	0.51494	.00177*	0.00004	1001000
	5	15	0.73333	0.48046	.08553	0.00027	1001000
	6	15	0.13333	0.12874	1.00000	0.00000	1001000
	7	15	0.20000	0.28736	.32547	0.00045	1001000

Genot, genotypes; Obs.Het., observed heterozygosity; Exp.Het., expected heterozygosity; *SD*, standard deviation.

Tests for HWE for each locus within each population used an HWE test analogous to Fisher's exact test. *p* values were obtained using Arlequin ver 3.5. *Significant *p *≤* *.05.

## 4 DISCUSSION

Haplotype studies related to Hb G‐Coushatta in American, Chinese, Thai and Turkish individuals suggest a multiple origin for this variant (Itchayanan et al., 1999; Li et al., 1999; Ozturk et al., 2007). Previously published β‐globin gene cluster haplotypes data in association with the Hb G‐Coushatta cases (American Indian [− + – – + – ?], Chinese [– + + – + + ?], Kocaeli‐Turkey [– + + – – – +] and Denizli‐Turkey [– + – + + + +]) support the prediction that this variant has a multi‐centric origin (Li et al., 1999; Ozturk et al., 2007). Interestingly, while the haplotype of [– + – + + + +] (Ozturk et al., 2007) obtained by pedigree analysis in the Denizli region was found to be in 4th place with 6% frequency (Table 1), the haplotype of [– + + – – – +] obtained by pedigree analysis performed with the samples in the Kocaeli region is not included in the diversity of haplotypes list obtained from the Denizli region Hb G‐Coushatta population in this study (Table 1). Similarly, American Indian [– + – – + – ?], and Chinese [– + + – + + ?] types are not on the haplotype list associated with the Hb G‐Coushatta mutation in the Denizli region (Table 1). The fact that the American, Chinese and Kocaeli‐Turkish type haplotypes are not among the haplotypes associated with Hb G‐Coushatta mutation in the Denizli region supports the literature view on the independent and multi‐centric origin of this mutation. The haplotype diversity and frequency percentages in Tables 1 and 2 show that the Hb G‐Coushatta mutation most probably developed on the normal population gene pool in Denizli, Turkey. The haplotypes representing the different genetic origins of the Kocaeli and Denizli Hb G‐Coushatta populations which are geographically close regions support the view that the Hb G‐Coushatta mutation is independent from the historical migration routes.

Table 3 summarizes the results of the AMOVA test statistic calculated the degree of genetic differentiation between normal and Hb G‐Coushatta populations in the Denizli region. These results indicate that negligible genetic differentiation (5.96%) between the two populations (*F*_ST_: 0.05964, *p *=* *0.01369 ± 0.00367) (Table 3). This low and statistically significant (*p < *.05) genetic differences showed that the two populations are not diversified by the effect of migration on the gene pool. The difference in haplotype diversity between the two populations may be the possible effect of mutation formation on polymorphic loci in the β**‐**globin gene cluster.

Table 4 showed the high and similar haplotypic diversity (*h*), low nucleotide diversity (π) and similar average number of pairwise nucleotide differences (*k*) between the two populations (Li, 1997; Nei, 1987). Additionally, Fu's *F*_s_ statistic showed a significant negative value for the two populations, indicating similar population expansion throughout history for these populations. Tajima's *D* values were insignificant (*p *>* *.05) for the two populations, suggesting that these populations are at neutral equilibrium (Table 4). These findings indicate that the molecular diversity of both populations have genetically similar development and expansion in the historical period (Fu, 1997; Tajima, 1989a,b, 1993). The mismatch distribution parameters in Table 4 were investigated using mismatch distribution analysis to estimate the demographic developments of the two populations (Harpending, 1994). According to the graphs obtained from the calculated distribution parameters, the normal population appeared to be unimodal (*unimodal distribution; the exponential growth is smooth*), while the Hb G‐Coushatta population is departure from the unimodal distribution (Figure 1; *gray bar*). The reason for this difference in distribution is that the Hb G‐Coushatta population presented in Table 6 is in departure from Hardy–Weinberg Equilibrium (HWE) of the fourth locus (Guo & Thompson, 1992). HWE (*p *>* *.05) means that there will be no change in allelic or genotypic frequencies from one generation to the next. However, with the possible effect of Hb G‐Coushatta mutation in the fourth locus may have occurred as the difference between the observed and expected pairwises. Mismatch distribution results were supported by the level of Harpending's raggedness index and *p* values of SSD in Table 5 (Harpending, 1994; Ozturk et al., 2016).

Our results suggested that the origin of the Hb G‐ Coushatta population in Denizli province may have been in the Mediterranean area, separated from Hb G‐Coushatta population in Kocaeli region which geographically close region and other populations rather than from recent Asiatic migrations. According to our estimated values of τ show that the average time since the demographic expansion for normal and Hb G‐Coushatta populations ranged from approximately 42,000 ybp (95% CI; 11,000–55,000) and 38,000 ybp (95% CI; 10,000–62,000), respectively. Historic demographic expansions were investigated by the examination of frequency distributions of pairwise differences between sequences (mismatch distribution), which is based on three parameters: θ_0_, θ_1_ (θ before and after the population growth) and τ (time since expansion expressed in unit of mutational time) (Table 4) (Rogers & Harpending, 1992). In our published studies, the average time since the demographic expansion of Hb S and Hb D‐Los Angeles populations in Denizli was calculated as range from approximately 26,000 ybp (95% CI; 11,000–36,000) and 38,000 ybp (95% CI; 18,500–62,000), respectively (Ozturk et al., 2016, 2017).

According to the Klein's results, *Homo sapiens neanderthalensis* (HN) constitute a group of hominids whose particular morphology developed in Europe during the last 350,000 years under the effect of selection and genetic drift reaching its final form approximately 130,000 ybp (Klein, 2003). This subgroup of hominids populated the Europe and Western Asia approximately 45,000 ybp, until the arrival of *Homo sapiens sapiens* (HS), the first modern humans (Mellars, 1992; Parker, 1993). This is an available data on European mtDNA diversity indeed support this view. The most European populations present a signal of Paleolithic demographic expansion from a small population, which could be dated to about 40,000 ybp (Excoffier & Schneider, 1999). Entrance of Homo sapiens to Europe was between 50,000 to 46,000 ybp. Today most Europeans can trace their ancestry by mtDNA lines that appeared among 50,000 and 13,000 ybp (Oppenheimer, 2012).

Our results indicate that the Hb G‐Coushatta population was not introduced into the Anatolian gene pool by migration from Asia or any other geographical region, compatible with the published dating results. Since Asiatic tribal migrations were recent events (about 2 000 ybp) we had to observe the genetic drifts in our data but we did not observe genetic drifts during the time course of about 40,000 ybp up to the present time.

In conclusion, these findings further suggest that the Hb G‐Coushatta population originated in the normal population in Denizli, Turkey. Although two populations share common genetic origin findings, they have different haplotype variations. We think that the reason for these variations is the departure from Hardy–Weinberg equilibrium of the fourth locus in the Table 6. The possible effect of the Hb G‐Coushatta mutation on polymorphic loci in the β‐globin gene cluster may cause this haplotypic variation between normal and Hb G‐Coushatta populations.

## CONFLICT OF INTERESTS

Authors Ozturk, Arikan, Atalay and O. Atalay declare that they have no conflicts of interest.

## AUTHOR CONTRIBUTIONS

SA, provided support in the data generation of the laboratory results, sample collection and preparation. AA and EOA supervised the study, support in the data interpretation and manuscript preparation.
